# The effect of water-soluble tomato concentrate on elevated serum cholesterol in the middle-aged and elderly Chinese individuals

**DOI:** 10.3389/fnut.2024.1410420

**Published:** 2024-09-11

**Authors:** Yingxiang Yu, Yifan Wu, Lan Xie, Cuiqing Chang

**Affiliations:** ^1^Department of Sports Medicine, Peking University Third Hospital, Beijing, China; ^2^Institute of Sports Medicine, Peking University, Beijing, China; ^3^Beijing Key Laboratory of Sports Injuries, Beijing, China

**Keywords:** water-soluble tomato concentrate, blood pressure, elevated serum cholesterol, hypertension, hypercholesterolemia

## Abstract

Water-soluble tomato concentrate (WSTC) has demonstrated beneficial effect on blood flow in healthy populations. The prospective, randomized, double-blind, and placebo-controlled clinical trial was conducted to explore the impact of WSTC on individuals with elevated cholesterol levels. Sixty participants aged 35–65 with high cholesterol were enrolled and evenly divided into a treatment group (FFG) and a placebo group (PCG). Over a 60-day period comprising a 45-day treatment phase followed by a 15-day observational follow-up. Participants in the FFG received 300 mg daily of Fruitflow tablets, while the PCG were received placebos. The study showed that there were no significant differences in baseline parameters between the FFG and PCG (*p* > 0.05). Post-intervention, the FFG exhibited significant reductions in systolic blood pressure (SBP) and diastolic blood pressure (DBP) by 4.2% (SBP, *p* < 0.001) and 3.8% (DBP, *p* = 0.015), respectively, compared to the PCG (*p* = 0.041). These reductions were sustained during the follow-up period. In contrast, the PCG showed no significant changes in SBP and DBP (*p* > 0.05). Stratified analysis by hypertension status revealed a significant SBP reductions both hypertensive and non-hypertensive FFG subjects (*p* < 0.05), with a trend towards DBP reduction. No significant changes in SBP and DBP were observed in the PCG. Moreover, the FFG group showed a significant increase in high-density lipoprotein (HDL) cholesterol (*p* < 0.05), along with a marked reduction in both weight and body mass index (BMI) (*p* < 0.05). The FFG also showed decreased levels of homocysteine, high-sensitivity C-reactive protein, and fasting blood glucose compared to the PCG (*p* < 0.05). In conclusion, WSTC has the potential to lower blood pressure and cardiovascular risk profiles in hypercholesterolemic individuals, presenting a viable non-harmacological option for enhancing cardiovascular health.

**Clinical trial registration**: https://www.chictr.org.cn/showproj.html?proj=27052, identifier ChiCTR1800015904.

## 1 Introduction

The increasing prevalence of cardiovascular disease (CVD) in China correlates with a surge in modifiable risk factors, positioning CVD as the leading cause of mortality and a critical public health dilemma (1). Investigations highlight dyslipidemia, particularly hypercholesterolemia, as a significant contributor to thrombogenesis, leading to the development of atherothrombosis, which manifests as myocardial infarction and stroke (2, 3). Accordingly, cholesterol mitigation is central to the strategic management of atherosclerotic cardiovascular disease (ASCVD), aiming to attenuate this escalating healthcare burden.

Dietary natural products have shown significant promise in preventing cardiovascular disease due to their multifaceted benefits, including the improvement of endothelial function, effective cholesterol management, reduction of low-density lipoprotein (LDL) levels, elevation of high-density lipoprotein (HDL) levels, potent anti-inflammatory activities, and antioxidant properties (4–6). Notably, tomatoes are highlighted, with epidemiological evidence linking increased consumption to reduced cardiovascular risk (7). The emerging interest in tomato’s hydrophilic compounds, including flavonoids, phenolic acids, and tannins, has highlighted their antioxidant, anti-inflammatory, and anti-thrombotic properties, essential for endothelial health (8, 9). However, further research is imperative to clarify the cardioprotective efficacy of these bioactives in varied populations, aiming to provide an evidence-based foundation for dietary contributions to health optimization and precision in cardiovascular disease prevention.

Water-soluble tomato concentrate, known as WSTC, extracted from mature tomatoes and primarily composed of adenosine, flavonoids, and chlorogenic acid, has garnered significant interest among researchers (10–12). Some studies have shown that WSTC positively influences hemodynamics in healthy individuals without negatively affecting coagulation mechanisms (10, 13, 14). Furthermore, recent research suggests that WSTC may have a potential therapeutic role in lowering blood pressure in males with prehypertension (15). Drawing from these research insights, our study is designed as a double-blind, randomized controlled trial to examine the potential impacts and action mechanisms of WSTC on individuals with elevated cholesterol, regarding blood pressure, lipid profiles, Homocysteine (Hcy) levels, and body composition. This approach aims to provide a more substantial scientific basis for the application of natural food bioactives in the prevention and treatment of hypercholesterolemia and its related health risks.

## 2 Methods

### 2.1 Participant recruitment

Individuals with elevated serum cholesterol levels were included in this study, which was approved by the Peking University Third Hospital Medical Science Research Ethics Committee (No. [2017]325–03) and registered at the China Clinical Trial Registration Center (Registration No. ChiCTR1800015904). All participants were informed of the purpose, content, and risks involved with study participation; written informed consent was collected prior to the commencement of the trial.

### 2.2 Inclusion and exclusion criteria

*Inclusion criteria:* (1) Cholesterol: Total Cholesterol (TC) ≥ 5.2 and < 7.2 mmol/L, and/or Low-Density Lipoprotein Cholesterol (LDL-C) ≥ 3.4 and < 4.9 mmol/L; (2) Age: 35–65 years; (3) Cholesterol elevation without prior or recent lipid-lowering treatment.

*Exclusion criteria:* (1) Platelet count <100 × 10^9/L; (2) Coagulation disorders; (3) Recent use of aspirin or other platelet/coagulation modifiers; (4) Consumption of fish oil or evening primrose oil supplements within the last month; (5) Regular consumption of tomatoes and their products (≥ 5 days/week); (6) Tomato allergy; (7) Pregnancy or lactation; (8) Major cardiovascular, hepatic, renal, or endocrine disorders; (9) Non-consent.

### 2.3 Study design and intervention

The study was conducted as a prospective, randomized, double-blind, placebo-controlled clinical trial design. Sixty participants were sequentially allocated random codes by an independent third party. The study lasted for 60 days, including a 45-day intervention period followed by a 15-day follow-up. Throughout the intervention phase, participants were administered the trial product, which was either WSTC or a placebo, orally with meals at a dose of 300 mg twice daily. Administration was discontinued during the follow-up period. Each 300 mg tablet of WSTC contained 150 mg of Fruitflow, including flavonoids, adenosine, and chlorogenic acid, sourced from DSM (China) Co., Ltd. The remaining half of the tablet weight was made up of excipients, such as microcrystalline cellulose, lactose, and magnesium stearate, and was coated with a sugar film. The placebo consists of the same excipients as Fruitflow (including microcrystalline cellulose, lactose, and magnesium stearate), formulated into 300 mg tablets and sugar-coated. Its shape, size, color, and taste are indistinguishable from Fruitflow tablets.

### 2.4 Outcomes and measurements

Assessment and Measurement of Relevant Indices at Pre-Intervention (Day 0), Post-Intervention (Day 45), and End of Follow-Up (Day 60).

#### 2.4.1 Clinical outcomes

Blood pressure was measured using an Omron electronic sphygmomanometer after a 10-min rest in a quiet room, averaging two readings. Blood lipid profiles, including TC, Triglycerides (TG), LDL-C, HDL-C, high-sensitivity C-reactive protein (hs-CRP), and FBG were analyzed using a biochemical analyzer (Beckman Coulter 5,800, United States). Homocysteine (Hcy) levels were determined via electrochemiluminescence with an automated chemiluminescence immunoassay analyzer (Abbott IM2000, United States). Body composition, including weight, BMI, and BF%, was measured with a body composition analyzer (MC-180, Tanita Corporation; Japan).

#### 2.4.2 Safety parameters

These include complete blood count, coagulation profile, and liver and kidney function tests.

#### 2.4.3 Adverse events

Throughout the trial period, participants were required to record any related adverse symptoms.

### 2.5 Evaluation of physical activity and nutritional intake

Physical activity is quantified using the Chinese version of the International Physical Activity Questionnaire (iPAQ) Short Form (16), with weekly activity levels expressed in MET-minutes/week. Dietary intake of vegetables and fruits is assessed through the Food Frequency Questionnaire (FFQ), and daily energy intake is calculated according to the China Food Composition (2nd Edition).

### 2.6 Statistical analysis

Randomization is conducted by non-research personnel, with study implementation and data collection handled by the research team. Blinding is lifted post-study, and analysis is performed solely on data from participants who completed the study. Normal distribution data are presented as mean ± standard deviation (*x*ˉ ± *s*), while non-normal data are expressed as median (interquartile range) M (P25, P75). Group comparisons for parametric data use independent sample t-tests, and paired t-tests for within-group temporal comparisons. Non-parametric data employ rank-sum tests. Analyses are conducted using SPSS 23.0, with two-sided tests and a significance threshold set at *p* < 0.05.

## 3 Results

### 3.1 Baseline characteristics

**Figure 1:**
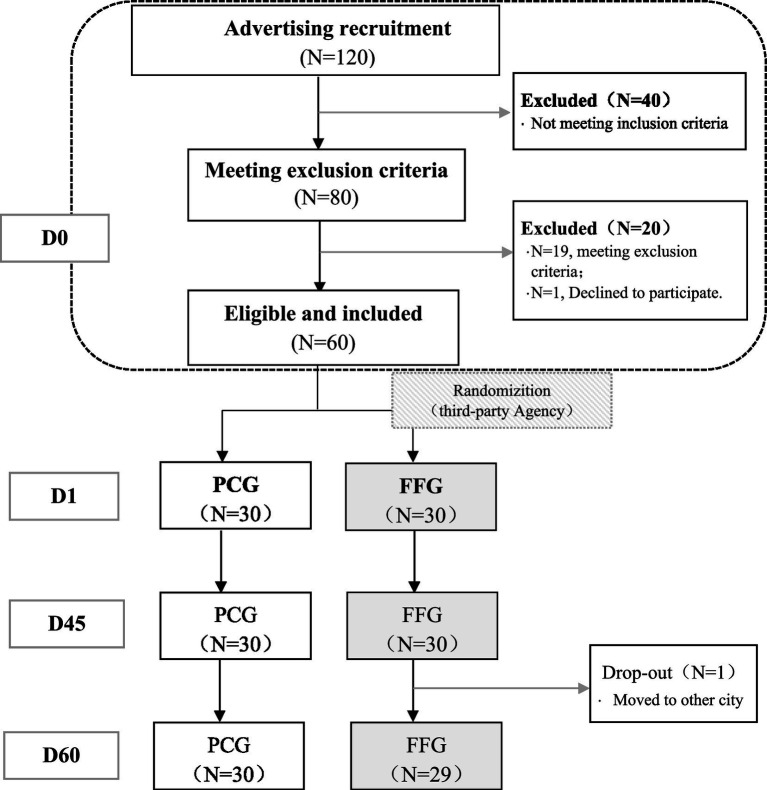
Participant flow chart. D0 is the period from informed consent to just before D1 (within 2 weeks). D1 is the trial start (medication onset). D45 is the last day of the intervention. D60 is the final day, marking the end of follow-up period.

The study enrolled 60 participants, evenly divided with 30 in the Fruitflow Group (FFG) and 30 in the Placebo Control Group (PCG). All 60 subjects completed the intervention, with 59 completing the follow-up (as illustrated in Figure 1). Detailed demographic and baseline characteristics, presented in Table 1, show no significant differences between the FFG and PCG across measured indicators (*p* > 0.05).

**Table 1:** General baseline characteristics of patients (***x̄*** ±*s*).

	PCG	FFG	*p* value
*N*	30	30	
Age (years)	54.7 ± 6.4	51.5 ± 7.2	0.122
Sex (male/female)	6/24	5/25	0.807
Height (cm)	161.7 ± 7.6	159.7 ± 8.7	0.355
Body weight (kg)	66.6 ± 11.0	63.9 ± 12.2	0.245
BMI (kg/m^2^)	25.5 ± 3.8	24.6 ± 3.0	0.311
SBP (mmHg)	127.8 ± 13.0	125.9 ± 14.7	0.599
DBP(mmHg)	76.5 ± 10.4	78.5 ± 9.1	0.424
^TC (mmol/L)	6.01 (5.60, 6.44)	5.71 (5.46, 6.47)	0.745
LDL-C (mmol/L)	4.05 ± 0.66	4.03 ± 0.89	0.906

BMI, Body weight index; SBP, systolic blood pressure; DBP, Diastolic blood pressure; TC, Total Cholesterol; LDL-C, Low-Density Lipoprotein Cholesterol; FFG, Fruitflow Group; PCG, Placebo Control Group. ^expressed as M (P25, P75).

### 3.2 Changes in blood pressure

In the FFG, a 4.2% reduction in systolic blood pressure (SBP) was observed after 45 days of intervention (*p* < 0.001), which was significantly lower than in the PCG (*p* = 0.041). The follow-up period showed a 1.5% increase in SBP (*p* = 0.032), yet it remained below the baseline level. Diastolic blood pressure (DBP) decreased by 3.8% (*p* = 0.015). In contrast, no significant changes in SBP and DBP were observed in PCG during the intervention and follow-up. Detailed data can be found in Table 2.

**Table 2:** Change in blood pressure (***x̄*** ±*s*).

	Groups	Total			Hypertensive			Non-hypertensive		
		*N*	SBP (mmHg)	DBP (mmHg)	*N*	SBP (mmHg)	DBP (mmHg)	*N*	SBP (mmHg)	DBP (mmHg)
Before intervention	PCG	30	127.8 ± 13.0	76.5 ± 10.4	10	132.2 ± 12.8	79.7 ± 11.63	20	125.6 ± 12.9	74.8 ± 9.6
	FFG	30	125.9 ± 14.7	78.5 ± 9.1	7	138.6 ± 15.6	85.0 ± 5.5	23	122.8 ± 11.9	76.5 ± 9.2
	*p* value		0.599	0.424		0.371	0.281		0.362	0.563
Post-intervention	PCG	30	128.2 ± 14.6	76.9 ± 10.2	10	132.1 ± 12.4	80.7 ± 9.3	20	126.3 ± 15.5	75.0 ± 10.3
	FFG	30	120.6 ± 13.4^*^	75.5 ± 8.0^*^	7	132.4 ± 12.1^*^	80.0 ± 6.2	23	117.0 ± 11.8^*^	74.2 ± 8.1
	*p* value		0.041	0.566		0.957	0.865		0.033	0.770
Follow-up	PCG	30	127.4 ± 15.1	78.9 ± 9.2	10	133.9 ± 9.9	84.0 ± 8.1	20	124.0 ± 16.5	76.2 ± 8.8
	FFG	29	124.3 ± 13.5△	78.0 ± 8.1	6	137.7 ± 11.2△	83.7 ± 4.9	23	120.8 ± 11.9△	75.5 ± 6.6
	*p* value		0.413	0.696		0.512	0.925		0.473	0.904

* Compared to before intervention，*p* <0.05; △compared to post-intervention; *p* < 0.05. SBP, systolic blood pressure; DBP, Diastolic blood pressure; FFG, Fruitflow Group; PCG, Placebo Control Group.

Further stratified analysis based on hypertension status revealed significant reductions in SBP in FFG after the 45-day intervention (*p* < 0.05), regardless of hypertension status, with a minor decrease in DBP (*p* > 0.05). In non-hypertensive FFG subjects, SBP was significantly lower than that in controls. In PCG, no significant changes in SBP or DBP were observed during the intervention and follow-up. Detailed findings are presented in Table 2.

### 3.3 Changes in lipid profile

After a 45-day intervention, the FFG showed a significant increase in HDL-C by 6.5% (*p* = 0.018). However, levels of TG, TC, and LDL-C did not change significantly. In the PCG, there were no significant changes observed in these lipid parameters. Additionally, no significant differences were observed between the groups. Detailed information is provided in Table 3.

**Table 3:** Change in cardiovascular disease-related risk factors.

	Groups	*N*	TG^	TC^	LDL-C^#^	HDL-C^	hs-CRP^	Hcy^	FBG^
			(mmol/L)	(mmol/L)	(mmol/L)	(mmol/L)	(mg/L)	(mmol/L)	(mmol/L)
Before intervention	PCG	30	1.54 (1.07, 2.39)	6.01 (5.60, 6.44)	4.05 ± 0.66	1.33 (1.16, 1.68)	1.04 (0.66, 2.04)	10.51 (8.16, 14.31)	5.90 (5.37, 6.42)
	FFG	30	1.46 (1.15, 2.60)	5.71 (5.46, 6.47)	4.03 ± 0.89	1.22 (1.05, 1.41)	0.85(0.40, 1.97)	9.44 (8.30, 11.04)	5.55 (5.20, 6.12)
	*p* value		0.626	0.745	0.906	0.101	0.271	0.212	0.169
Post-intervention	PCG	30	1.45 (1.10, 2.40)	5.71 (5.41, 6.03)	3.77 ± 0.63	1.36 (1.15, 1.57)	1.23 (0.42, 3.22)	9.10 (8.42, 11.64)^*^	5.95 (5.20, 6.50)
	FFG	30	1.62 (1.11, 2.13)	5.87 (5.07, 6.42)	3.88 ± 0.78	1.41 (1.20, 1.47)^*^	1.05 (0.46, 1.84)	8.93 (6.97, 10.49)^*^	5.45 (5.20, 5.80)
	*p* value		0.959	0.893	0.545	0.824	0.391	0.322	0.173
Follow-up	PCG	29	1.47 (0.94, 2.10)	5.90 (5.21, 6.29)	3.87 ± 0.66	1.29 (1.16, 1.55)	1.11 (0.64, 2.79)	9.54 (8.16, 11.84)^*^△	5.70 (5.30, 6.45)
	FFG	30	1.48 (1.06, 2.37)	5.72 (5.08, 6.39)	3.73 ± 0.84	1.28 (1.09, 1.50)	0.67 (0.39, 1.59)	8.17 (6.69, 9.83)^*^△	5.40 (5.10, 5.72)
	*p* value		0.601	0.495	0.488	0.306	0.022	0.008	0.029

^*^Compared to before intervention, *p* < 0.05; △compared to post-intervention, *p* < 0.05; TG, Triglycerides; TC, Total Cholesterol; LDL-C, Low-Density Lipoprotein Cholesterol; HDL-C, high-density lipoprotein cholesterol; Hcy, Homocysteine; hs-CRP, high-sensitivity C-reactive protein; FFG, Fruitflow Group; PCG, Placebo Control Group. ^expressed as M (P25, P75); # expressed as (*x̄* ±*s*).

### 3.4 Changes in other risk factor-related indicators

As shown in Table 3, baseline levels of hs-CRP showed no statistical difference between the FFG and the PCG (*p* = 0.271). FFG exhibited a decreasing trend in hs-CRP levels, in contrast to an increasing trend in PCG. At the end of the follow-up, hs-CRP levels in FFG were significantly lower than those of PCG (*p* = 0.022). Both groups experienced significant reductions in Hcy levels during the intervention (FFG: -5.4%, *p* = 0.028; PCG: -13.4%, *p* = 0.006), with FFG continuing to decrease (−8.5%, *p* < 0.001) and PCG showing an increase (+4.8%, p < 0.001) at follow-up, resulting in significantly lower Hcy levels in FFG compared to PCG (*p* = 0.008). For FBG, no significant difference was observed between FFG and PCG at baseline (*p* = 0.169). Post-intervention, FBG levels decreased in FFG, while remaining stable in PCG. By the end of the follow-up, FBG levels in FFG were significantly lower than those of PCG (*p* = 0.029).

### 3.5 Changes in body composition

Post-intervention and during the follow-up, both body weight and BMI showed significant reductions in the FFG and displayed statistical differences compared to the PCG (*p* < 0.05). The PCG exhibited no significant changes in weight and BMI throughout the intervention and follow-up periods (*p* > 0.05). Changes in body fat percentage (BF%) were not significant in either group. Details are provided in Table 4.

**Table 4:** Changes in body composition (***x̄*** ±*s*).

	Group	*N*	BW (kg)	BMI (kg/m^2^)	BF% (%)
Before intervention	PCG	30	66.65 ± 10.98	25.48 ± 3.78	33.39 ± 7.11
	FFG	30	63.89 ± 12.22	24.61 ± 3.01	30.78 ± 6.92
	*p* value		0.360	0.325	0.154
Post-intervention	PCG	30	66.71 ± 10.77	25.49 ± 3.65	32.53 ± 7.08
	FFG	30	63.24 ± 12.50^*^	24.34 ± 3.10^*^	30.63 ± 6.87
	*p* value		0.254	0.194	0.295
Follow-up	PCG	29	66.86 ± 11.02	25.40 ± 3.75	33.05 ± 6.69
	FFG	30	63.14 ± 12.60^*^	24.44 ± 3.09^*^	30.71 ± 6.94
	*p* value		0.236	0.291	0.197

^*^Compared to before intervention, *p* < 0.05. BW, body weight; BMI, body weight index; BF%, percentage of body fat; FFG, Fruitflow Group; PCG, Placebo Control Group.

### 3.6 Physical activity and dietary intake

Neither the FFG nor the PCG showed significant changes in physical activity levels during the intervention and follow-up periods (*p* > 0.05). Total energy, vegetable, and fruit intakes remained consistent across both groups throughout these periods (*p* > 0.05).

### 3.7 Safety evaluation

Before and after the intervention, coagulation, liver, and kidney function parameters remained within normal ranges for participants in both groups.

## 4 Discussion

### 4.1 Antihypertensive efficacy of Fruitflow and possible mechanisms

Our results indicate that Fruitflow effectively decreases blood pressure levels in individuals with hypercholesterolemia, particularly SBP, demonstrating partially sustained efficacy. This significant impact on blood pressure regulation is one of the key highlights of our research. During the intervention period with Fruitflow, notable reductions in both SBP and DBP were observed, with these lower levels being maintained for 15 days post-intervention. Notably, the effect of Fruitflow on reducing blood pressure was observed regardless of the presence of hypertension. Existing research supports our hypothesis that the antihypertensive action of Fruitflow is intrinsically linked to its rich composition of flavonoids, adenosine, and chlorogenic acid.

Based on this, studies have shown that flavonoid-rich plant extracts act as natural inhibitors of Angiotensin-Converting Enzyme (ACE), thereby functioning as natural ACE inhibitors (ACEIs) (17). Specifically, chlorogenic acid, classified as a phenolic acid within the polyphenol category, has been demonstrated to inhibit and downregulate ACE and renin expression (18). This effect contributes to the synthesis of endothelial Nitric Oxide (NO) (19), inducing vasodilation and consequent blood pressure reduction. Furthermore, adenosine, known for its role in immune and inflammatory pathways, can mitigate various inflammatory disorders, thus modulating vascular inflammatory damage (20). Complementing these findings, research by Biswas et al. (21) has revealed that WSTC inhibits ACE activity in rat lungs. Based on these insights, we propose that Fruitflow may exert its antihypertensive effects through anti-inflammatory mechanisms, by enhancing endothelial function, increasing NO release, and reducing ACE and renin levels. Therefore, this study provides substantial clinical evidence supporting Fruitflow’s role in blood pressure management, particularly for hypercholesterolemic populations. However, the precise underlying mechanisms of these effects warrant further detailed investigation.

### 4.2 Effect of Fruitflow on lipid profiles and other cardio-cerebrovascular risk factors

Apart from its anti-hypertensive properties, our study reveals multiple beneficial effects of Fruitflow in individuals with elevated cholesterol. These effects include: (i) A significant increase in HDL-C levels indicates that Fruitflow is beneficial for reducing the risks of cardiovascular diseases. Since HDL-C is inversely correlated with the risk of coronary heart disease, its beneficial roles extend beyond reverse cholesterol transports (22, 23). These include anti-inflammatory and antioxidant properties, thrombosis inhibition, and the enhancement of endothelial function, collectively mitigating arteriosclerosi. (ii) Reduction in FBG levels. Elevated FBG is a key contributor to arteriosclerosis, primarily due to mechanisms such as advanced glycation end-product formation, insulin resistance, and polyol metabolism abnormalities, leading to endothelial dysfunction, oxidative stress, and the release of pro-inflammatory cytokines, thus accelerating arteriosclerosis (24). (iii) Decrease in hs-CRP levels. As a significant predictor of cardiovascular diseases, high hs-CRP levels are involved in both localized and systemic inflammatory responses, impairing endothelial cells, reducing NO and prostaglandin release, upregulating Angiotensin I receptors, and affecting renin and angiotensin levels, which contribute to endothelial cell proliferation, intimal thickening, and arteriosclerosis progression (25, 26). (iv) Notable reduction in Hcy levels, with this effect persisting for 15 days post-intervention. Hyperhomocysteinemia (HHcy) is associated with oxidative stress, endothelial dysfunction, arterial stiffening, and angiotensin activation (27). Epidemiological studies in Chinese populations have demonstrated HHcy’s significant role in increasing hypertension risk (28, 29) and as an independent risk factor for the one-year recurrence of acute ischemic stroke (30). (v) Substantial reduction in both body weight and BMI. Therefore, Fruitflow exhibits a protective role against arteriosclerotic cardiovascular diseases, achieved by elevating ‘good cholesterol’ levels and reducing FBG, hs-CRP, Hcy, body weight, and BMI, thus proving beneficial for cardiovascular health.

### 4.3 The safety of Fruitflow

After the intervention with Fruitflow, there were no abnormalities in the coagulation function, liver function, and kidney function of the subjects, and no adverse events occurred during the trial period, indicating that Fruitflow is safe.

In summary, this study indicates that Fruitflow effectively lowers blood pressure in individuals with hypercholesterolemia and positively modulates cardiovascular risk factors, with sustained effects. The limited sample size and brief duration of the intervention in this study may lead to certain underestimations. Specifically, the small sample size could underestimate diet’s impact on the study outcomes. Future studies would benefit from a larger sample size to enable stratified randomization of potential confounding factors. Moreover, the short duration of the trial might result in underestimate the long-term effects of Fruitflow on cardiovascular health, highlighting the need for a longer intervention period. Overall, these improvements will provide a robust scientific basis for using Fruitflow in the precise management of hypertension and contribute to developing novel approaches for utilizing bioactive food components in the prevention and management of cardiovascular diseases.

## Data availability statement

The raw data supporting the conclusions of this article will be made available by the authors, without undue reservation.

## Ethics statement

The studies involving humans were approved by Peking University Third Hospital Medical Science Research Ethics Committee. The studies were conducted in accordance with the local legislation and institutional requirements. The participants provided their written informed consent to participate in this study.

## Author contributions

YY: Writing – original draft, Data curation, Investigation, Methodology, Resources, Writing – review & editing. YW: Data curation, Formal analysis, Software, Validation, Visualization, Writing – review & editing. LX: Investigation, Methodology, Resources, Writing – review & editing. CC: Conceptualization, Funding acquisition, Project administration, Supervision, Writing – review & editing.
